# Impact of Low Inclusion Rate of Olive Cake in Dairy Cow Rations on Uterine Health and Fertility Indices During Early Lactation

**DOI:** 10.3390/ani15020269

**Published:** 2025-01-19

**Authors:** Ioannis Nanas, Themistoklis Giannoulis, Eleni Dovolou, Ilias Giannenas, Georgios S. Amiridis

**Affiliations:** 1 Department of Obstetrics and Reproduction, Veterinary Faculty, University of Thessaly, 43100 Karditsa, Greece; gnsnanas@gmail.com; 2 Laboratory of Genetics, Department of Animal Science, University of Thessaly, 41223 Larissa, Greece; thgianno@uth.gr; 3 Laboratory of Reproduction, Department of Animal Science, University of Thessaly, 41223 Larissa, Greece; entovolou@uth.gr; 4 Laboratory of Nutrition, Faculty of Veterinary Medicine, School of Health Science, Aristotle University of Thessaloniki, 54124 Thessaloniki, Greece; igiannenas@vet.auth.gr

**Keywords:** dairy cows, olive cake, fertility, acute phase proteins, immune system, gene expression, uterine cytology

## Abstract

Olive cake was incorporated at a low inclusion rate (3.7%) into the rations of dairy cows through partial substitution of maize, and its effects on milk production, general health, and fertility traits were investigated. Multiparous purebred Holstein dairy cows (n = 148) were divided into two groups: a treated group (n = 86) and a control group (n = 62). The control ration (group C) was modified by replacing 1 kg of maize with an equal quantity of olive cake (group T). The experiment lasted from 60 days prepartum to 150 days postpartum. Electronic aids were utilized to quantify daily milk yield and detect estrus expression, while clinical and uterine examinations were performed weekly. Blood samples, uterine epithelial cells, and endometrial tissue samples were collected from a subgroup of healthy animals in both groups at specific time points. Blood samples were examined to determine the β-hydroxybutyric acid (BHBA), acute phase proteins (haptoglobin [Hpt] and serum amyloid A [SAA]), progesterone, and pregnancy-associated glycoproteins concentrations. Uterine epithelial cells were assessed for polymorphonuclear neutrophil (PMN) counts, and the expression of nine genes encoding inflammatory cytokines and immune system activation was analyzed in uterine biopsy tissue. No significant differences (*p* > 0.05) were observed between groups in milk yield, general morbidity, clinical endometritis, or conception rates. However, animals in group T came to estrus approximately 6 days earlier (*p* = 0.013) than those in group C; progesterone concentrations on day 7 of the subsequent cycle tended (*p* = 0.07) to be higher in group T. On day 21, BHBA concentrations were higher in group C than in group T (*p* < 0.05). Throughout the experiment, Hpt levels in group T were consistently lower (*p* < 0.001), while SAA was lower on day 7 compared to group C. From days 21 to 42 postpartum, there was a significant reduction in PMN numbers (group C *p* = 0.02; group T *p* < 0.0001), with a tendency for a greater reduction rate in group T (*p* = 0.08). Among the genes studied, a significant difference was revealed in the expression of the ILA1 gene, with strong correlations of gene expression in group C and weak to moderate correlations in group T. In conclusion, under the conditions of this experiment, the inclusion of olive cake into dairy cows’ rations did not affect milk production but improved certain health and fertility parameters, making olive cake a suitable alternative feedstuff for high-producing animals.

## Simple Summary

Olive oil is the utmost ingredient in the Mediterranean diet, offering numerous health benefits to consumers. However, the extraction of olive oil generates significant by-products, which pose serious environmental challenges. The dehydrated residue, known as olive cake, has been utilized as an alternative feedstuff for various farm animals due to its low cost and rich content of polyphenols and unsaturated fatty acids, which provide antioxidant and anti-inflammatory properties. Additionally, it contributes to environmental sustainability and aligns with the principles of a circular economy. In this study, maize was replaced with olive cake in the diets of dairy cows, and various parameters including milk yield, overall health, uterine health, and fertility were assessed. Although no significant difference in milk production was observed, cows fed olive cake exhibited an earlier onset of postpartum estrus, improved health, and more rapid restoration of the uterine environment post-calving compared to control cows. These findings suggest that incorporating olive cake into dairy cow diets is not only environmentally beneficial but also enhances overall health and fertility.

## 1. Introduction

Olive oil production constitutes the largest segment of the Greek primary sector, with an annual output of approximately 300,000 tons. According to the Eurostat (https://ec.europa.eu/eurostat/web/main/search accessed on 28 November 2024 ) and the International Olive Council (https://internationaloliveoil.org, assessed on 28 November 2024) in Greece, the olive trees (*Olea europaea*) are cultivated in 750,000 agricultural holdings, occupying more than 1 million hectares of arable land. With this production, Greece ranks third worldwide after Spain and Italy. Since 2005, the annual global olive oil consumption has been steadily increasing, which puts strong pressure on the intensification of production. However, each ton of olive oil produced generates a corresponding amount of waste, which can be equal to or up to twice the quantity of oil extracted, depending on the extraction method. The olive oil by-products contain fruit pulp, skin, stones, leaves (18–32%), and water (40–60%), which pose a serious environmental hazard for the production areas. Traditionally these wastes have been used as fertilizers, or fuel by households, greenhouses, and local cheese dairies. In recent years, the partly dehydrated olive cake has been incorporated into farm animal diets. This practice has been shown to provide multiple benefits, including the reduction in environmental pollution and minimized management costs for production units, while the cake can partly substitute the inclusion rate of expensive cereals or forages in animals’ rations. On a dry matter basis, the olive cake contains proteins (6–8%), fatty acids (5–9%), cellulose (7–9%), lignin (26–30%), hemicellulose (7–9%) and various phenolic compounds, such as tyrosol, hydroxytyrosol, and oleuropein. Its energy content is comparable to that of cereals [1,2]. Notably, only a very small proportion (0.5 to 2%) of the olive fruit phenolic compounds remains in the olive oil, while, at least 98% is retained in the olive cake [3]. Depending on the method of extraction, the total phenolic compound of the olive cake ranges from 2.2 gr/kg to 4.4 gr/kg [4]. The unsaturated fatty acids and the presence of phenols confer unique properties to olive cake, allowing it to be characterized as a potential bioactive or functional feedstuff. Olive oil contains various biocomponents with antioxidant, free radical scavenging, and anti-inflammatory properties. Its high inclusion rate in the Mediterranean diet is linked to the relatively low incidence of various types of cancer, atherosclerosis, and coronary heart disease (reviewed by [5,6,7]). Phenolic compounds are present in nearly all plants and fruits; however, olives are recognized as a significant natural source of bio-phenols, leading to the incorporation of olive products into numerous pharmaceutical and cosmetic formulations. The addition of olive cake to animal diets is increasingly attractive due to its positive impact on nutrition costs and its contributions to improved antioxidant and anti-inflammatory status, as well as potentially enhancing the immune status of the animals [8]. Further, the exploitation of olive cake as an alternative feedstuff contributes to addressing an alarming environmental issue related to the excessive generation of waste. At the same time, it improves the nutritional properties of milk by increasing the concentration of monounsaturated fatty acids (MUFAs) and decreasing the saturated fatty acids (SFAs) levels [2], of cheese by decreasing the concentration of de novo synthesized saturated fatty acids and the atherogenic index and increasing the monounsaturated long-chain fatty acids in the Haloumi [2,9], and of meat by increasing the tocopherol concentration in muscles and improving the overall oxidative stability of the meat [10]. It is noteworthy that improved product properties do not possess any adverse effects on the overall productivity of the animals [2,9,10].

The transition period for dairy cows—defined as 21 days before and after calving—is characterized by a physiological need for cows to adapt from a low nutrient demand during the dry period to the high energy requirements of early lactation. During this period, the incidence of metabolic or infectious diseases is at its peak among all productive phases of the animal’s life. Disorders that occur during this transition can impair milk production and future fertility, increase the risk of early culling, and compromise cow welfare [11,12]. The negative energy balance (NEB) is regarded as a primary cause of increased morbidity and reduced production during early lactation [13,14,15]. While nutrition and NEB are directly related to metabolic disorders, they have a prominent role in infectious diseases, as they contribute to immune system functionality either directly through specific nutrients or indirectly through specific and biologically active metabolites [16]. Postpartum immunosuppression is a physiological condition, but the rigorous genetic selection for high milk production and the intensification of cattle production systems have been associated with the aggravation of this condition and subsequent reduction in fertility [17]. The role of the immune system is not confined to the defense mechanisms, but it is pivotal in many physiological processes, such as the detachment of the fetal membranes, the gamete transport, the corpus luteum formation, the maternal recognition of pregnancy, etc. [18]. It is, therefore, vital new dairy management practices are adopted to support undisturbed immune system functionality and fertility during the critical phases of cattle breeding.

In light of these considerations, we investigated the effects of feeding dairy cows with olive cake during the transition period and early lactation on reproductive performance, uterine health, and immunity in the early stages of lactation.

## 2. Materials and Methods

The procedures outlined in this study were approved by the Ethical Committee of Animal Welfare of the University of Thessaly Veterinary Faculty, (license number 112/19/9/20), and conducted in accordance with regulations pertaining to the protection of animals used for scientific purposes (Directive 2010/63).

### 2.1. Animals

The study was carried out on a commercial dairy farm in central Greece, which housed 380 Holstein cows, averaging a milk production of 9870 L/cow per lactation. Individual daily milk yield, rumination rate, and physical activity were electronically monitored and recorded via an automated electronic monitoring system installed in the farm (SCR, Cow Monitoring Systems, Allflex, Netanya, Israel). The cows of all groups were housed in sheltered loose pens equipped with cubicles having waterbed matrices bedding, at a 1 to 1 rate (1 cubicle per cow). An automated cooling system was installed including fans and sprinklers, which operated during the hot periods of the year. All animals were fed once daily a total mixed ration (TMR), had unlimited access to drinking water, and were milked three times daily at 5:00, 13.00, and 21:00.

The pregnant cows had a typical dry period lasting for 58 to 66 days prior to the expected calving, divided into two subgroups: the far-off (from drying off until day 18 to 21 pre-calving) and the close-up (18 to 21 days pre-calving) groups. The enrolled animals were selected from the far-off group and allocated into one of the two treatment subgroups of the close-up and lactation groups. Pregnant cows (n = 148) in their 2nd to 4th gestation were included in the study, which was conducted during the thermoneutral period of the year (October to April).

The control group comprised multiparous cows (group C, n = 62) with a mean parity of 3.0 ± 1.2 and a BCS of 3.6 ± 0.3. The treated group consisted of multiparous cows (group T, n = 86) with a mean parity of 3.4 ± 1.6 and a BCS of 3.8 ± 0.4. Animals in group C were fed with the standard ration used in the farm (TMR) based on forages, cereal grains, and soybean meal, according to the recommendations of INRA [19]. The modification of group T′s ration included the replacement of 1 kg of maize with an equivalent quantity of partially destoned dried olive oil cake (Ruminolive, Spartalife, Sparta, Greece). In the lactation ration, the inclusion rate was 3.7%; additional slight adjustments were made to ensure that both diets were isocaloric and isonitrogenous. The nutrient and chemical composition of the Ruminolive and the composition of the TMR of lactation are presented in Table 1 and Table 2, respectively. The modified ration was offered until day 150 post-partum (pp) at which point animals of both groups were combined into another lactation group.

### 2.2. Clinical Examinations

All clinical examinations were performed by the same veterinary surgeons who visited the farm biweekly. The pregnancy status of all animals was confirmed upon drying off and the body condition score (BCS) was evaluated on a five-point scale ranging from emaciation (score 0) to obesity (score 5) [20]. No clinical examinations were carried out during the entire dry period.

### 2.3. Post-Partum Uterine Examinations

During the first week of pp, all cows underwent a general clinical examination, and uterine content was evaluated via gloved hand sampling. In the presence of abnormal uterine content, the cows were treated accordingly. Cows diagnosed with metritis—characterized by an enlarged uterus with a red-brown watery, fetid vaginal discharge and an internal temperature of ≥39.5 °C—were treated with ceftiofur hydrochloride (2 mg/kg BW, Excenel, Zoetis, Louvain-la-Neuve, Belgium) and flunixin meglumine (2.2 mg/kg, Finixin, MSD, Berlin, Germany). Metricheck was performed after day 21 pp for the detection of clinical endometritis. Following disinfection of the vulval lips, the sterilized hemisphere of the instrument was lubricated and inserted into the vagina. The retrieved content was evaluated on a 5-point scale (0 to 4), where: 0 = healthy cows with clear and transparent mucus; 1 = no pus but clouded mucus (indicating mild endometritis); 2 = mucus with pus traces; 3 = mucus containing <50% mucopurulent material; 4 = discharge with >50% pus and/or sanguineous mucus. Cows scored as 2 or 3 were treated with two PGF2α injections (0.5 µg cloprostenol, 2 mL, Estrumate, MSD, Friesoythe, Germany) given with an interval of 11 to 14 days. The same PGF2α treatment along with an intrauterine cephapirine (500 mg/cow, Metricure, MSD, Bad Waldsee, Germany) treatment given 5 days after the first prostaglandin injection was applied to cows with a score of 4. Two weeks after treatment, all treated cows were re-examined to assess uterine health or determine if additional treatment was necessary.

### 2.4. Ovarian Resumption, Estrus Expression, Artificial Insemination and Pregnancy Diagnosis

Estrus detection was based on physical activity data provided by the electronic monitoring system and was confirmed by visual observation of the sexual behavior of the cow. Ovarian examinations commenced in the third-week pp. The day pp at which a corpus luteum (CL) was identified by rectal palpation/ultrasonographic examination was defined as the time of ovarian resumption; this was subsequently confirmed by the first rise in blood progesterone concentration above 1.5 ng/mL.

The voluntary waiting period was set at 65 days in milk. Any cow that had not expressed estrus by day 60 pp was synchronized with the double OVSYNCH protocol [21]. All cows were inseminated with semen from the same sire by the same farm technician.

Pregnancy-associated glycoproteins (PAG) concentrations were determined on days 29 to 36 post A.I. for initial pregnancy diagnosis, which was confirmed 10 to 15 later by rectal palpation and/or transrectal ultrasonography.

### 2.5. Hematological and Biochemical Examinations

On days −7, 0, 7, 14, 21, and 42 (calving = day 0), blood samples were collected from the tail vein in plain tubes for the analysis of acute phase protein concentrations. The samples were allowed to clot and serum was separated by centrifugation and stored at −20 °C until analysis for haptoglobin (Hpt) and serum amyloid (SAA).

Acute phase proteins were determined in blood samples collected on days −7, 7, and 21 from healthy animals without any sign of clinical metritis and being scored Metricheck 0 or Metricheck 1 on days 14 and 21 (n = 10 and n = 16 for groups C and T, respectively), using commercially available ELISA Kits (MyBioSource, San Diego, CA, USA). The Hpt assay sensitivity was 0.02 ng mL^−1^, and intra- and inter-assay coefficients of variation were 5.1% and 5.7% for samples containing low concentrations and 4.5% and 7.7% for high concentrations. SAA assay had a sensitivity of 0.1 ng mL^−1^, while the intra- and inter-assay coefficients of variation for samples with low and high concentrations were 5.9%, 7.5%, 9.5%, and 6.7%, respectively.

On days 7, 14, and 21, blood glucose and BHBA levels were determined by cow-side tests (Precision Xseed, Abbott, CA, USA) using the respective glucose and ketone strips.

Progesterone concentrations were analyzed weekly starting from day 21 pp until the expression of overt standing estrus. In addition, progesterone concentration was determined on day 7 in 10 control and 20 treated cows. The hormone assessment was performed by a commercial ELISA kit (NRG, Marburg, Germany). The sensitivity of the assay was 0.08 ng mL^−1^, while the intra- and inter-assay coefficients of variation for low- and high-concentration samples were 5.4%, 6.8%, 9.7%, and 5.6%, respectively.

Pregnancy-associated glycoprotein (PAG) concentration was analyzed by a commercial ELISA (DG29 kit, Conception Animal, Beaumont, QC, Canada) with an assay sensitivity and specificity of 96.8% and 98.7%, respectively [22]. All samples were analyzed in duplicates and any sample with >5% difference within the duplicate was re-assayed.

### 2.6. Cytology

Endometrial cytological samples were collected from six animals in each group that had previously been scored as Metricheck 1 on days 21 and 42 pp, following the methodology described by [23]. Briefly, after disinfecting the vulva and perineum, a cytology brush (Andwin Scientific, Simi Valley, CA, USA) was attached to a sterile metal stem and entirely covered with a sterile stainless-steel tube. This system was introduced transcervically into the uterine body, and the stem was advanced firmly to allow the brush to protrude from the tube. The metal stem was rotated two or three times before being retracted into the tube. The brush was then rolled onto a glass slide. The smear was allowed to dry, fixed with methanol, stained with Giemsa, and stored until microscopic evaluation at magnifications of ×100 to ×400.

Each slide was examined blindly by the same operator, and the mean of two examinations was used for analysis. Endometrial cells, epithelial cells, and polymorphonuclear cells (PMNs) were counted, with a total of 300 cells evaluated. The percentage of PMNs was calculated as their number divided by the sum of PMNs and epithelial cells [24,25].

### 2.7. Uterine Biopsy

Uterine biopsies were performed on days 35 to 37 pp on the same animals that had been previously sampled for cytological examination. Following the application of caudal epidural anesthesia (4–5 mL of Lidocaine, Xylosan Demo SA, Athens, Greece), the vulva and perineum were disinfected. Laparoscopic biopsy punch forceps (R. Wolf 8383.124, 5 mm × 33 cm, Knittlingen, Germany), covered with a protective plastic sheath, were guided to the external orifice of the cervix; the sheath was ruptured by pulling it backward, allowing the instrument to be introduced into the uterus and advanced approximately 1 cm into the right uterine horn. The jaws of the instrument were opened, and the uterine wall was firmly pressed into the opened jaws. The endometrial sample was obtained by closing the jaws of the instrument before its removal. The tissue sample was extracted using a sterilized hypodermic needle and stored at −20 °C in RNA later until further processing.

### 2.8. RNA Extraction and cDNA Synthesis

Total RNA was extracted using E.Z.N.A. (Omega Bio-tek Inc, Norcross, GA, USA). Total RNA Kit I, according to the manufacturer′s protocol. An extra step of DNAse treatment was performed after the extraction to ensure the removal of any DNA residues. The Maxima First Strand cDNA Synthesis kit for RT-qPCR was used for the synthesis of complementary DNA (cDNA) following the manufacturer’s protocol.

### 2.9. Gene Expression Analysis

The genes of the study are coding for immune system proteins, since they participate in essential immunity pathways, namely interleukin signaling, cell recruitment, TLR signaling, etc. (Data from Reactome Database). Each gene was designed using Primer3 (https://primer3.ut.ee/ accessed on 17 March 2024) and was checked at Beacon Designer to ensure their conformational suitability (http://www.premierbiosoft.com, accessed on 17 March 2024). Last, UCSC In-Silico PCR (https://genome.ucsc.edu/cgi-bin/hgPcr, accessed on 17 March 2024) was used to ensure the unique annealing location of the primers in the cow genome. Furthermore, a set of 3 housekeeping genes were used (YWHAZ, SDHA, and GUSB), to normalize the gene expression values. The genes and the respective primers are shown in Table 3. GeNorm and M value as an indicator of the gene expressions’ stability across samples were used to evaluate the suitability of the reference genes.

Real-time PCR (qPCR) was performed using the SYBR Green Technology with a Rorot-Gene Q (Qiagen, Hilden, Germany). Analysis of qPCR was carried out in a 10 μL reaction volume after the addition of 1.5 μl cDNA in the PCR mix containing gene-specific primers (200 nM final concentration) and 1x KAPA SYBR FAST qPCR Master mix (Sigma-Aldrich, Burlington, MA, USA). qPCR conditions were 5 min at 95 °C and 40 cycles of 20 s at 95 °C and 20 s at 60 °C for annealing and extension. At the end of every reaction, a melt curve analysis was performed to ensure the specificity of the products. Samples were measured in duplicate and a maximum ± 0.2 difference in Cq values was applied as a threshold in the duplicates’ measurements.

For the differential gene expression analysis between the groups, a combinatorial LinReg–Quantification Cycle (Cq) approach was used: Cq values were retrieved for each reaction by setting a constant threshold, and the average efficiencies per gene were computed using the LinReg software (version 2021.2). The relative gene expression was normalized using the geometric mean of the three reference genes.

### 2.10. Statistical Analysis

The morbidity rate between groups was analyzed by a chi-square test. The days to first estrus were analyzed by student’s *t*-test.

The statistical analyses for the biochemical markers (BHBA, glucose, progesterone, and acute phase proteins) and PMNs as well as for the data from the Metricheck examinations were performed in R, using the appropriate packages and tests. A normality test was performed to check deviations from the normal distributions in order to choose the appropriate parametric or non-parametric test. For the Metricheck data, a Fisher exact test was performed, and for the PMN data, a repeated measures ANOVA analysis was applied to detect the effect of two factors (feed and day) on the levels of PMNs, followed by a posthoc test for pairwise comparisons between groups. A statistical threshold of *p*-value < 0.05 was applied in all analyses.

The statistical analysis of DEGs was performed using R (version 4.3.2) and more specifically the following:

1. The Shapiro test was applied in each gene to test for deviations from the normal distribution and select the appropriate statistical tests for the differential gene expression.
2. The Wilcoxon test statistic among the groups fed with different was computed using the function wilcox.test for each gene (threshold *p* < 0.05).
3. Spearman correlation coefficients were computed for each pair of genes in each group using the cor function since correlated gene expression may be indicative of a similar regulation mechanism underlying gene expression.
4. Boxplots and correlation coefficient plots for the gene expression data were designed using ggplot2 (version 3.5.1).

## 3. Results

### 3.1. Milk Yield

No difference (*p* = 0.75) was recorded in the milk production during the first 100 days of lactation (4073 ± 778 kg and 3980 ± 827 kg for groups C and T, respectively).

### 3.2. General and Uterine Health

The chi-square test revealed that during the study period, both groups had similar (*p* = 0.61) clinical disease incidence. The following numbers of clinical cases were recorded in groups C and T, respectively: retained placenta 3 and 4, abomasal displacement 1 and 1, metritis 3 and 3, ketosis 2 and 3, milk fever 1 and 1, lameness 1 and 1, endometritis 5 and 6, and mastitis 7 and 11.

**Figure 1:**
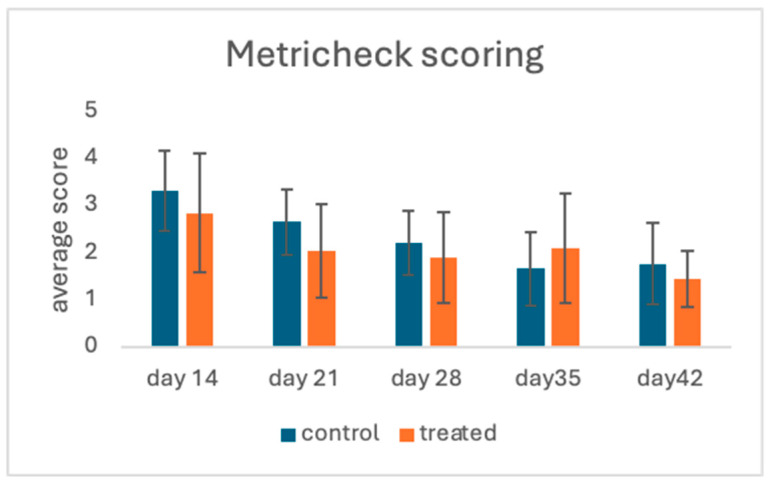
Metricheck scoring between groups on different days.

In both groups, the average Metricheck score declined in a time-dependent manner. Between days 14 and 42, a reduction rate of almost 50% was recorded in both groups. Although no statistically significant difference was observed at any time point, in group T the mean score was steadily numerically lower than that of group C and tended to differ (*p* = 0.1) on day 28, (Figure 1).

### 3.3. Estrus Expression, Progesterone, and Conception Rate

Until day 60 pp, 38 (61.3%) group C and 59 (68.6%) group T cows were detected in standing heat (*p* > 0.05), by electronic signaling, visual observation, and in all cases by increased progesterone levels. For these animals, the calving to first estrus interval was shorter (*p* = 0.013) in group T (40.1 ± 10.5 days) compared to group C (46.1 ± 11.1 days).

On day 7, after the expression of the spontaneous estrus, progesterone concentration tended (*p* = 0.07) to be higher in animals of group T (6.60 ± 1.09 ng/mL) compared with group C (6.02 ± 0.86 ng/mL).

No difference (*p* > 0.05) was observed in the percentage of pregnant cows by day 120 pp (71.0% and 76.7%, for groups C and T, respectively).

### 3.4. Glucose and BHBA

Glucose concentrations ranged from 28 mMol/L to 93 mMol/L; at any time point, no difference (*p* > 0.1) was detected between the animals of the two groups.

**Figure 2:**
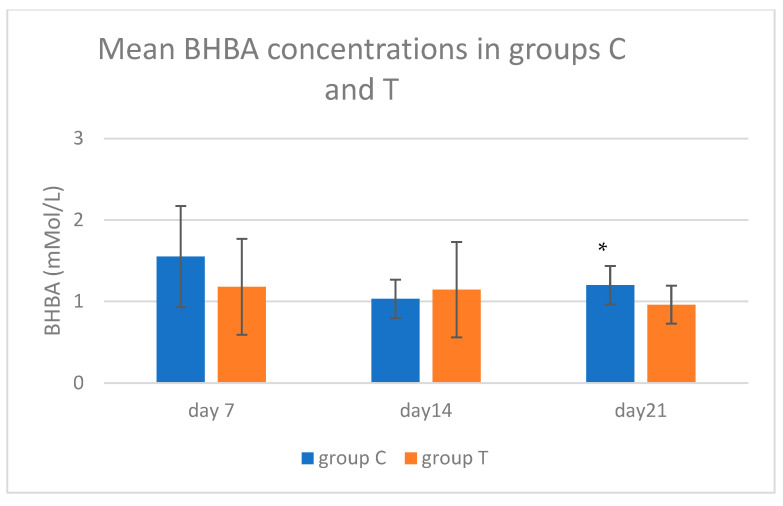
Mean BHBA concentrations on different sampling days in control and treated groups. Asterisk (*) denotes statistical difference (*p* < 0.05).

On day 21, BHBA concentration in group T cows (0.96 ± 0.23 mMol/L) was lower (*p* = 0.041) than in group C (1.20 ± 0.24 mmol/L). Data on BHBA concentrations are shown in Figure 2.

### 3.5. Acute Phase Proteins

**Figure 3:**
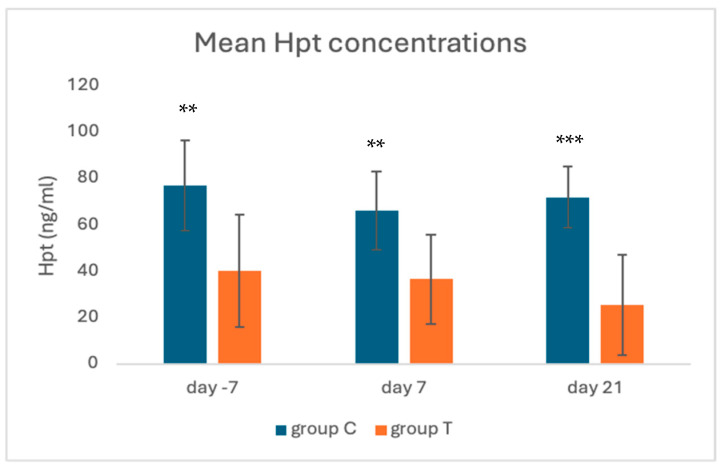
Mean Hpt concentrations in groups C and T, on different sampling days. ** *p* < 0.001, *** *p* < 0.0001.

**Figure 4:**
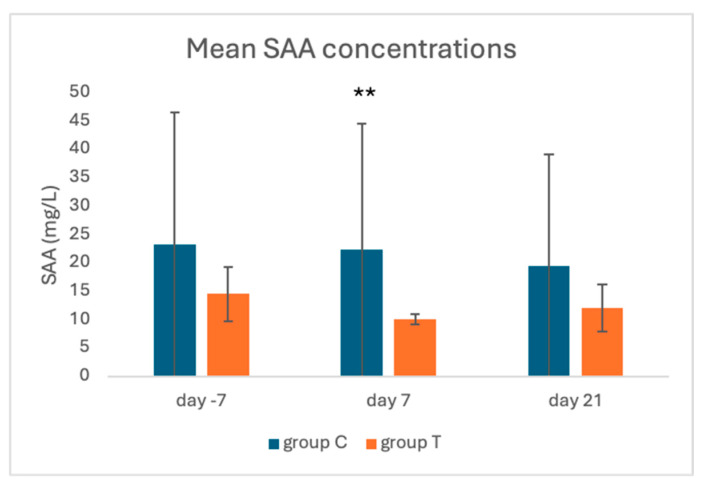
Mean SAA concentrations in groups C and T, on different sampling days. ** *p* < 0.001.

Significant differences were detected in the concentrations of both acute phase proteins between groups; details are shown in Figure 3 and Figure 4. At all time points, Hpt concentrations were lower (*p* < 0.001) in group T animals compared to group C (day −7 40.5 ± 24.3 ng/mL and 77.2 ± 19.5 ng/mL; day 7, 36.7 ± 19.3 ng/mL and 66.4 ± 16.8 ng/mL; day 21, 25.6 ± 21.5 ng/mL and 72.2 ± 13.4 ng/mL, for groups T and C, respectively).

Compared to group C, SAA levels in group T were lower (*p* = 0.0003) on day 7 and tended (*p* = 0.062) to be so on day −7, while no difference was detected on day 21 (day −7 14.6 ± 4.8 mg/L and 23.3 ± 15.0 mg/L; day 7, 10.1 ± 0.9 mg/L and 22.3 ± 16.7 mg/L; day 21, 12.1 ± 4.1 mg/L and 19.6 ± 12.0 mg/L, for groups T and C, respectively).

### 3.6. Uterine Cytology

**Figure 5:**
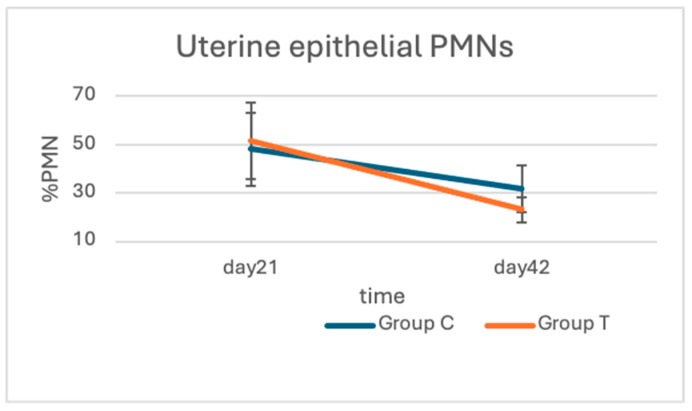
Mean PMN rates on different sampling days in groups C and T.

The two-way ANOVA analysis revealed that time affected the mean PMN numbers (*p* = 0.0014). No difference was detected in the mean PMNs between groups in days 21 and 42. Between the two time points, the mean PMN (%) differs within groups (group C, 48.0 ± 14.9 and 31.8 ± 9.6, *p* = 0.02, and group T, 51.5 ± 15.9 and 23.1 ± 5.8, *p* < 0.0001, for days 21 and 42, respectively). The reduction rates (%) between days 21 and 42 were 32.1 ± 13.3 and 50.6 ± 21.5 (*p* = 0.08) for groups C and T, respectively (Figure 5).

### 3.7. Differential Gene Expression and Correlation Coefficients

**Figure 6:**
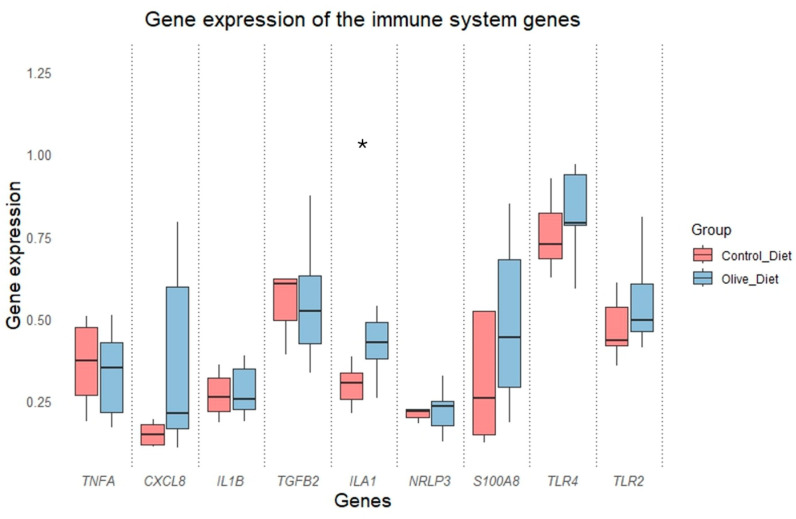
Relative gene expression of the genes under study in both groups. The expression in pairs of genes marked with asterisk (*) differ significantly (*p* < 0.05).

Among the nine genes under study, only one gene showed differential gene expression between the two groups, ILA1, which exhibited an upregulation in the olive diet, and CXCL8 showed a tendency towards differential gene expression between the groups, being upregulated in the olive diet. The corresponding *p*-values are shown in Table 4 and the respective boxplots are shown in Figure 6.

**Figure 7:**
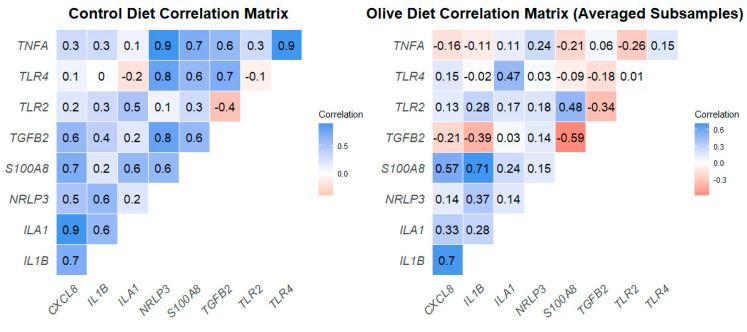
Correlation graphs of gene expression for the two diets.

Correlation coefficients showed differences between the two diets, as shown in Figure 7. In group C, there were only positive correlation coefficients, with specific pairs of genes exhibiting strong positive correlations (Table 5). On the other hand, the correlations in group T were weak or moderate, and, in contrast to the control diet, there were also negative correlations (Figure 7).

## 4. Discussion

The exploitation of compounds with high biological value is undoubtedly where the olive oil sector should be directed. While the health benefits of olive oil consumption for humans are well established, it is equally important to highlight the potential advantages for animal health and farm profitability through the inclusion of olive oil by-products in animal rations. Evidence is accumulating that olive oil by-products are valuable sources of bioactive compounds that can be recovered using green technologies and incorporated into animal feed in alignment with circular economy strategies. The anticipated benefits of such practices include a reduction in feeding costs, an enhancement of the nutritional quality of milk produced, and the conservation of cereals for human consumption. However, these alternative feeds must not only provide immediate economic benefits but also ensure the long-term health and productivity of the animals. Therefore, a thorough investigation into their biological effects on animal physiology is essential prior to their incorporation into dairy cow diets. Olive cake is one such alternative feedstuff, and its effects on farm animals have been extensively studied by research groups from Mediterranean countries, which are the primary, if not the sole, producers of this product [2,26].

In the present study, the inclusion rate was set at 3.7% on a DM basis, which is considered relatively low. This decision was made after pre-experimental observations indicated that a 7% inclusion rate led to prolonged customization periods during which the dry cows exhibited reduced dry matter intake. During the first 100 days postpartum, no significant difference in milk yield was observed between control cows (group C) and those fed olive cake (group T). This finding aligns with other studies reporting neutral effects of various olive oil by-products (including olive cake, partly destoned cake, and olive paste silage) on milk production, even at inclusion rates as high as 15% of DM (IR 5% [9], IR 5.6% [27], IR 10% [28], IR 13% [29], and IR 15% [30,31]).

Although the effects of olive cake have been extensively studied in relation to milk yield and milk quality characteristics, to the best of our knowledge, this is the first study to report on reproductive indices and uterine health parameters after the inclusion of olive cake in dairy cows’ rations.

Cows in group T exhibited earlier expression of estrus and had lower concentrations of β-hydroxybutyric acid (BHBA) on day 21 pp compared to group C. The transition period and the initial months of lactation are characterized by significant changes in metabolism and immune system functionality that are interrelated and directly associated with increased morbidity. During early lactation, cows mobilize fat reserves to support milk synthesis; while this is a normal adaptive process, excessive fat mobilization due to severe negative energy balance can affect the immune system and increase the risk of metabolic and infectious diseases [12]. Conversely, metabolic status and energy balance are critical determinants for the timely restoration of communication among the components of the hypothalamic-pituitary-ovarian axis. BHBA results from partial hepatic oxidation of non-esterified fatty acids (NEFA) produced during fat mobilization in periods of heightened energy demand [32]. Cows that resume ovarian activity early typically experience mild or no negative energy balance and demonstrate improved fertility compared to those with delayed luteal activity [33,34]. Early ovarian resumption is associated with shorter calving-to-conception intervals and overall higher pregnancy rates [35,36]. While the role of energy availability in controlling postpartum ovarian resumption is well recognized, the dietary energy availability in this study did not differ among groups; therefore, any observed differences should be attributed to factors other than energy density or availability.

Lactogenesis, which commences before parturition and peaks a few weeks thereafter, is accompanied by increased lipolysis and markedly reduced lipogenesis, contributing to a decrease in adipose mass [37]. Excessive lipolysis is considered an inflammatory process that activates macrophages [38] and induces oxidative stress characterized by the excessive production of free radicals such as reactive oxygen and nitrogen species [39]. Throughout the transition period, particularly immediately after calving, the increase in milk synthesis and immune system activation necessitates a high energy supply in the form of glucose [40]. During inflammatory responses, an overstimulated immune system withholds significant amounts of glucose, exacerbating existing negative energy balances [41]. This is manifested by elevated NEFA levels and subsequently high BHBA concentrations [42]. In our study, the increased BHBA concentrations observed in the control cows can be interpreted as a result of enhanced lipolytic and inflammatory responses, as indicated by persistently elevated levels of haptoglobin (Hpt) and serum amyloid A (SAA) on day 7. During bacterial or viral infections or under stress, monocytes secrete pro-inflammatory cytokines (IL-1, IL-6, TNF-α). Similarly, during the periparturient period, increased lipolysis accompanied by elevated pro-inflammatory cytokines suggests a condition resembling sterile inflammation or metabolic syndrome in humans [43,44]. Pro-inflammatory cytokines bind to specific receptors on hepatocyte membranes and induce an acute phase response that includes the liver’s release of acute phase proteins [45]. Both Hpt and SAA are positive acute-phase proteins that increase during the acute-phase response [46]. It is well established that ketosis, characterized by elevated concentrations of non-esterified fatty acids (NEFA) and beta-hydroxybutyric acid (BHBA), is positively correlated with increased levels of acute phase proteins (APPs) [47,48]. Notably, in group C, but not in group T, the BHBA concentrations on day 21 indicated mild subclinical ketosis, reflecting a higher negative energy balance. Therefore, we propose that the elevated BHBA levels are positively associated with increased acute phase protein concentrations in group C, or that the more pronounced subclinical conditions in this group contributed to the sustained elevation of acute phase proteins. It is important to note that in the present study, the animals assessed for acute phase protein concentrations and polymorphonuclear cell (PMN) counts were clinically healthy, exhibiting only very mild uterine infections that, according to standard procedures, required no treatment. Between days 21 and 42 postpartum, the number of PMNs in both groups decreased; however, both the degree of difference and the clearance rate were greater in group T, suggesting an enhanced anti-inflammatory capacity in these animals. This effect may be attributed to the biological properties of the polyphenols present in olive cake. Similar findings we observed in a previous study where soymeal was substituted with a mixture of flaxseed and lupins rich in polyunsaturated fatty acids [23]. However, other researchers argue that elevated PMN counts may simply indicate enhanced cellular migration rather than serve as a definitive marker of inflammation [49,50].

Based on PMN counts and the Metricheck score, both groups exhibited mild endometritis at the initial examination (day 21). Current understanding posits that PMNs constitute a critical first line of defense against pathogens and are vital components of the innate immune response. These cells migrate from the vascular compartment to sites of inflammation to kill or phagocytize bacteria [51], but they can also contribute to the pathogenesis of conditions characterized by disturbed tissue homeostasis, such as trauma and shock [52]. The earlier restoration of ovarian resumption in group T denotes that these animals were exposed to high estrogen levels earlier than those of group C. It is well established that estradiol stimulates vascularization of the endometrium [53,54], enhances uterine contractility [55], initiation of sexual receptivity [56], and supports sexual-specific effects on the secretory immune system [57]. It is, therefore, logical to assume that, at least partly, the faster clearing of the endometrium in group T could be attributed to the early exposure to estrogens.

Olive cake is recognized as an excellent source of polyphenols, and numerous studies support the beneficial properties of these compounds. For instance, Vergani et al. [58] demonstrated that polyphenols extracted from olive pomace protect hepatocytes against excess fat and oxidative stress. The biological activity of polyphenols and lignans derived from olive oil by-products has been investigated in relation to their cardioprotective effects within the Mediterranean diet [59]. Additionally, olive oil phenolics have shown beneficial effects on oxidation in vitro; for example, polyphenols have been found to reduce reactive oxygen species (ROS) production while exhibiting significant free radical scavenging properties [60,61]. In humans, antioxidant activity in the blood increases following the consumption of olive oil phenolic compounds [62]. Compounds with antimicrobial properties may help inhibit the growth of microorganisms and serve as therapeutic agents in the treatment of certain infectious diseases [63]. Phenolic compounds found in olive cake, including hydroxytyrosol, tyrosol, and oleocanthal, have demonstrated potent activity in vitro against bacteria responsible for intestinal and respiratory infections [64]. Hydroxytyrosol, which is abundant in olive cake, has been reported to exert significant anti-inflammatory effects in animal models by attenuating the expression of pro-inflammatory cytokines such as TNF-α and interleukin-1 beta (IL-1β) [65]. Furthermore, an in vitro study indicated that hydroxytyrosol reduces levels of inducible nitric oxide synthase (iNOS), cyclooxygenase-2 (COX-2), and TNF-α in lipopolysaccharide (LPS)-challenged human monocytic THP-1 cells [66]. Olive polyphenols have also been shown to mitigate inflammatory responses in a carrageenan-challenged mouse model of inflammation [67]. In trying to interpret our findings related to uterine health, we infer that in group T animals the early exposure to high estrogen concentrations along with lower degree of fat mobilization and enhanced immune function, facilitated resolution of the uterine contamination more effectively compared to group C. This hypothesis is further supported by the results from Metricheck assessments, the cytology, and gene expressions. As shown in Figure 6, the group T exhibited a significant upregulated expression of *IL1A* and a strong tendency towards upregulation of *CXCL8. IL1A* is a member of the cytokine family, which acts as a mediator of the immune response, while *CXCL8* encodes for a member of a chemokine family, acting as a chemotactic factor by guiding the neutrophils to the site of infection (data from NCBI). Both genes were found to be upregulated in bovine endometrial epithelial cells in vitro when they were infected with strains of Streptococcus uberis [68]. Additionally, the expression of *IL1A*, as well as *CXCL8*, was found upregulated in cows suffering from postpartum endometritis [69,70]. Thus, the over-expression of these genes may be involved in the restoration of uterine contamination, despite the reduction in the number of PMNs in the T group. Thus, we propose that the inclusion of olive cake in the diets of dairy cows enhances the ability of the uterus to resolve existing mild inflammation through the anti-inflammatory and antioxidant properties of the polyphenolic compounds present in the feed.

Cows experiencing negative energy balance may exhibit suppressed pulsatility of luteinizing hormone (LH), which adversely affects the ovarian response to LH [71]. This condition can lead to delayed ovarian resumption and the development of smaller ovulatory follicles, resulting in corpora lutea with diminished secretory capacity [35,72]. This was confirmed in the present study, where the progesterone concentration on day 7 of the estrous cycle in group C was lower than that in group T. Notably, cows in group T exhibited their first spontaneous postpartum estrus earlier than those in group C. Olive cake is a feed rich in polyunsaturated fatty acids, which influence the timing of follicular development, the first ovulation, and oocyte quality. Numerous studies have documented a strong positive correlation between the levels of polyunsaturated fatty acids and both the number of ovarian follicles and the size of the preovulatory follicle [73,74,75]. Additionally, higher concentrations of estradiol have been observed in cows fed diets rich in polyunsaturated fatty acids, with the accompanying granulosa cells demonstrating increased steroidogenic capacity, indicative of healthy follicle development [75,76].

In the context of this experiment, no statistically significant difference was detected in the pregnancy establishment rate by day 120 postpartum; however, the pregnancy rate in group T was 5.7% higher than in group C. This lack of significant difference may be attributed to the limited number of observations. For monotocous species such as cattle, approximately 800 animals must be tested to demonstrate a 5% difference at *p* < 0.05 [77]. Nevertheless, key parameters essential for restoring fertility and establishing early pregnancy were improved with the inclusion of olive cake in the diets of cows. The early resumption of ovarian function increased steroidogenic capacity of the corpus luteum and more rapid restoration of uterine health collectively provide a strong argument for the supportive role that olive cake can play in enhancing dairy cattle fertility, particularly in Mediterranean countries where this product is abundantly available in competitive prices that are usually lower than that of cereals.

## 5. Conclusions

The findings of this study provide compelling evidence for the viability of utilizing dried by-products from the olive oil industry as a safe and cost-effective alternative to cereals. Incorporating olive cake into the diets of dairy cows presents a valuable opportunity for the olive oil sector to reduce the environmental impact associated with its waste products. The observed early postpartum ovarian resumption, enhanced steroidogenic capacity of the corpus luteum, decreased levels of acute phase proteins (APP), and effective resolution of subclinical endometritis strongly suggest that feeding olive cake to high-producing cows supports immune system function, thereby improving health and fertility outcomes. The absence of statistically significant differences in conception rates is likely due to the relatively small sample size. Our results indicate a need for further research to optimize inclusion rates and explore the use of olive cake during immunosuppressive conditions, such as periods of summer heat stress.

## Figures and Tables

**Table 1:** Chemical composition of olive cake (Ruminolive).

Chemical Composition	Ruminolive
CP, %	8.50
EE, %	10.50
NDF, %	56.60
ADF, %	37.90
ADL, % *Fatty acid composition of olive cake *	14.0
Lauric acid (C12:0), %	0.05
Myristic acid (C14:0), %	0.05
Myristoleic acid (C14:1), %	11.09
Palmitic acid (C16:0), %	0.69
Palmitoleic acid (C16:1), %	0.19
Heptadecanoic acid (C17:0), %	0.09
Stearic acid (C18:0), %	2.71
Oleic acid (C18:1), %	70.77
Linoleic acid (C18:2) (ω-6), %	10.77
Linolenic acid (C18:3) (ω-3), %	0.48
Arachidic acid (C20:0), %	0.85
Eicosenoic acid (C20:1), %	0.11
Arachidonic acid (C20:4), % (ω-6)	0.13
Behenic acid (C22:0), %	0.12

CP = crude protein, EE = ether extract, NDF = nondetergent fiber, ADF = acid detergent fiber, ADL = acid detergent lignin.

**Table 2:** Ingredients and chemical composition of the experimental diets of lactating cows offered a standard ration (group C) or a modified ration with the addition of olive cake.

	Group C	Group T
Ingredient composition (kg)		
Maize silage	33	33
Alfalfa hay	2	2
Wheat straw	0.7	0.6
Grounded corn	4.0	5
Soybean meal (47%)	5.4	5.4
Cottonseed with lint	2.1	2.1
Molasses	1.4	1.4
Lipid supplement	0.25	0.17
Ruminolive	-	1
Sodium bicarbonate	0.15	0.15
Sodium chloride	0.03	0.3
Mineral-vitamin premix	0.5	0.5
*Chemical composition*		
DM, %	52.90	53.22
CP, % DM	18.08	17.93
EE, % DM	4.65	4.57
NFC, % DM	41.76	41.66
NDF, % DM	30.02	30.60
Ash, % DM	5.49	5.24
ME (Mcal/kg)	2527	2509

DM = Dry mater, CP = crude protein, EE = ether extract, NFC = non-fibrous carbohydrates, NDF = nondetergent fiber.

**Table 3:** List of genes under study and the designed primer pairs for each gene.

Gene Name	Gene ID	Gene Description	Forward Primer	Reverse Primer	Product Size (bp)
*CXCL8*	ENSBTAG00000019716	C-X-C motif chemokine ligand 8	CACATTCCACACCTTTCCAC	AAGCAGACCTCGTTTCCATT	80
*TLR2*	ENSBTAG00000008008	toll-like receptor 2	ACTGGACTGACTTTTCTTGAGG	TGGCTAATGTTCTGGATTGACT	88
*TGFB2*	ENSBTAG00000005359	transforming growth factor beta 2	CGGAGCGACGAGGAATAC	GTAGAAAGTGGGCGGGATG	176
*TLR4*	ENSBTAG00000006240	toll-like receptor 4	AGGTAGCCCAGACAGCATTT	GAGCGAGTGGAGTGGTTCA	77
*NLRP3*	ENSBTAG00000001273	NLR family pyrin domain containing 3	TAGGCAACAACGACTTGGGT	TCAGGCTTTTCAGGAGGCAG	80
*TNF*	ENSBTAG00000025471	tumor necrosis factor	CCCTTCTCATCCCCTTCTGG	GCCTCACTTCCCTACATCCC	79
*IL1B*	ENSBTAG00000001321	interleukin 1 beta	CCTCCGACGAGTTTCTGTGT	GCCAGCACCAGGGATTTTTG	79
*S100A8*	ENSBTAG00000062307	S100 calcium-binding protein A8	GAAGAAAAAGGATGCGGACA	TTATCACCAGCACGAGGAACT	92
*IL1A*	ENSBTAG00000047637	interleukin 1 alpha	CTGAGGCTACTATCTGTGGCT	CACGGCTTATTCCAACTGCT	105
*YWHAZ*	ENSBTAG00000046019	tyrosine 3-monooxygenase/tryptophan 5-monooxygenase activation protein zeta	CTGTAACTGAGCAAGGAGC	CCAAGATGACCTACGGGC	95
*GUSB*	ENSBTAG00000000236	glucuronidase beta	GCCGTTGTAATGTGGTCCTT	GGGCTTTAGTGTGGGCAATC	81
SDHA	ENSBTAG00000000704	succinate dehydrogenase complex flavoprotein subunit A	GCCCAGTGTGACCTCCTC	CATCATCAGAGCCCATCCCC	97

**Table 4:** Results of Wilcoxon test for each gene.

Gene	*p*-Value
*TNFA*	0.849
*CXCL8*	0.117
*IL1B*	0.633
*TGFB2*	0.703
*ILA1*	0.026
*NRLP3*	0.443
*S100A8*	0.289
*TLR4*	0.246
*TLR2*	0.289

**Table 5:** Strong correlations between pairs of genes in the control group.

Gene 1	Gene 2	Coefficient
*CXCL8*	*ILA1*	0.9
*NRLP3*	*TNFA*	0.9
*NRLP3*	*TGFB2*	0.8
*NRLP3*	*TLR4*	0.8
*TLR4*	*TNFA*	0.9

## Data Availability

Upon reasonable request, the corresponding author will provide the data supporting the results of the present study.
